# Data‐driven mapping of hypoxia‐related tumor heterogeneity using DCE‐MRI and OE‐MRI

**DOI:** 10.1002/mrm.26860

**Published:** 2017-08-30

**Authors:** Adam K. Featherstone, James P.B. O'Connor, Ross A. Little, Yvonne Watson, Sue Cheung, Muhammad Babur, Kaye J. Williams, Julian C. Matthews, Geoff J.M. Parker

**Affiliations:** ^1^ Division of Informatics, Imaging & Data Sciences The University of Manchester Manchester UK; ^2^ CRUK & EPSRC Cancer Imaging Centre in Cambridge and Manchester, Cambridge and Manchester UK; ^3^ Division of Cancer Studies The University of Manchester Manchester UK; ^4^ Department of Radiology Christie NHS Foundation Trust Manchester UK; ^5^ Division of Pharmacy & Optometry The University of Manchester Manchester UK; ^6^ Bioxydyn Ltd Manchester UK

**Keywords:** DCE‐MRI, OE‐MRI, cancer, clustering, heterogeneity, hypoxia

## Abstract

**Purpose:**

Previous work has shown that combining dynamic contrast‐enhanced (DCE)‐MRI and oxygen‐enhanced (OE)‐MRI binary enhancement maps can identify tumor hypoxia. The current work proposes a novel, data‐driven method for mapping tissue oxygenation and perfusion heterogeneity, based on clustering DCE/OE‐MRI data.

**Methods:**

DCE‐MRI and OE‐MRI were performed on nine U87 (glioblastoma) and seven Calu6 (non‐small cell lung cancer) murine xenograft tumors. Area under the curve and principal component analysis features were calculated and clustered separately using Gaussian mixture modelling. Evaluation metrics were calculated to determine the optimum feature set and cluster number. Outputs were quantitatively compared with a previous non data‐driven approach.

**Results:**

The optimum method located six robustly identifiable clusters in the data, yielding tumor region maps with spatially contiguous regions in a rim‐core structure, suggesting a biological basis. Mean within‐cluster enhancement curves showed physiologically distinct, intuitive kinetics of enhancement. Regions of DCE/OE‐MRI enhancement mismatch were located, and voxel categorization agreed well with the previous non data‐driven approach (Cohen's kappa = 0.61, proportional agreement = 0.75).

**Conclusion:**

The proposed method locates similar regions to the previous published method of binarization of DCE/OE‐MRI enhancement, but renders a finer segmentation of intra‐tumoral oxygenation and perfusion. This could aid in understanding the tumor microenvironment and its heterogeneity. Magn Reson Med 79:2236–2245, 2018. © 2017 The Authors Magnetic Resonance in Medicine published by Wiley Periodicals, Inc. on behalf of International Society for Magnetic Resonance in Medicine. This is an open access article under the terms of the Creative Commons Attribution License, which permits use, distribution and reproduction in any medium, provided the original work is properly cited.

## INTRODUCTION

Alongside the biological differences between tumors of different patients and within the same patient, there exists considerable functional and structural variation within individual lesions 1. This intra‐tumoral heterogeneity has been shown to be a marker of aggressive disease and poor patient prognosis 2, 3. Furthermore, the presence of certain phenotypical features in a tumor, such as hypoxia, is believed to contribute to therapeutic resistance 4, 5. Hypoxia can limit radiotherapeutic efficacy by reducing the radiosensitivity of tumor tissue and has also been shown to lessen the effect of chemotherapy 6. Drugs are being developed and tested that target hypoxic regions in tumors 7 with optimization and monitoring of treatment potentially benefiting from measuring intra‐tumoral hypoxia.

Clinically, characterization of the tumor microenvironment is carried out taking into account only limited spatial information. For hypoxia, voxel‐wise heterogeneity imaging is important to allow the detection of hypoxia in a background of normoxia. Oxygen tension probes 8, 9 and biopsies followed by histological staining 10, 11 are often treated as the “gold standard” for assessing oxygen tension in tissues. However, the sampled portion of tissue may not be representative of the entire tumor, biopsies of the same piece of tumor are non‐repeatable, and there may also be issues with accessing the tumor in some locations, such as in many lung cancers. These considerations necessitate a non‐invasive method for characterizing the tumor microenvironment and its heterogeneity, particularly one that is sensitive to hypoxia and has the potential to reliably predict and monitor treatment response.

Non‐invasive methods currently available for assessing inter‐ and intra‐tumoral oxygenation are largely positron emission tomography‐based or MRI‐based 5, 6. Positron emission tomography has a variety of radiotracers available such as [^18^F]MISO 12, [^18^F]FAZA 13, and [^64^Cu]ATSM 14, which have a high specificity for measuring hypoxia 15, although these more novel radiotracers are restricted to specialist centers with appropriately equipped radiochemistry departments. Clinically available methods of using MRI to assess tissue oxygenation 5 fall into those monitoring changes in $T_{2}^{*}$ relaxation using blood oxygenation level dependent (BOLD) contrast imaging 16 and those measuring changes in T_1_ relaxation using tissue oxygenation level‐dependent/oxygen‐enhanced (TOLD/OE)‐MRI 17. Their value in monitoring oxygenation in tumors has been demonstrated independently and in combination with each other 18, 19, 20, 21, 22, 23, 24, 25, 26. There are also studies that use dynamic contrast‐enhanced (DCE)‐MRI derived perfusion biomarkers, such as *K* ^trans^ and *AUC*_90_, as surrogate measures of hypoxia 27, 28, 29, 30.

We have previously carried out DCE‐MRI (reflecting tissue perfusion) and OE‐MRI (reflecting tissue oxygen delivery) on tumor xenografts, creating three classes of tumor sub‐regions based on enhancing/non‐enhancing voxels for both imaging techniques 31, 32. The class that enhanced with DCE‐MRI but not with OE‐MRI was termed the “perfused Oxy‐R fraction” (refractory to an oxygen challenge) and was postulated to represent hypoxic regions. Important steps have been taken toward providing biological and technical validation 33 of this biomarker for assessing the degree of tumor hypoxia.

Other workers have focused on objective, clustering‐based methods for locating distinct tumor (and other tissue) sub‐regions in general, without a specific focus on investigating hypoxia. This has been done in preclinical tumor models 34, 35, 36, 37, healthy kidneys 38, head and neck cancer 39, cervical cancer 40, bone metastases 41, and breast cancer 42. These studies demonstrate the use of data‐clustering techniques in segmenting tumor and healthy tissue, with varying levels of validation, but none present a systematic optimization of the parameters used in their methods.

The work we present in this paper investigates the application and optimization of data clustering methods such as those described above, but tailored and evaluated specifically on the combination of imaging data that we showed previously to map hypoxia: DCE‐MRI and OE‐MRI 21, 31. We present three ways of evaluating the performance of the clustering method and use these to select recommended parameters and options for the data sets in this study. We then compare our results with our previously published method to assess consistency of results and potential benefits of automated and objective tissue segmentation. We move away from a priori assumptions leading to heuristically chosen thresholds in data and present a method for mapping oxygenation and perfusion heterogeneity that is instead data‐driven and demonstrated to be robustly supported by the available signals.

## METHODS

**Figure 1:**
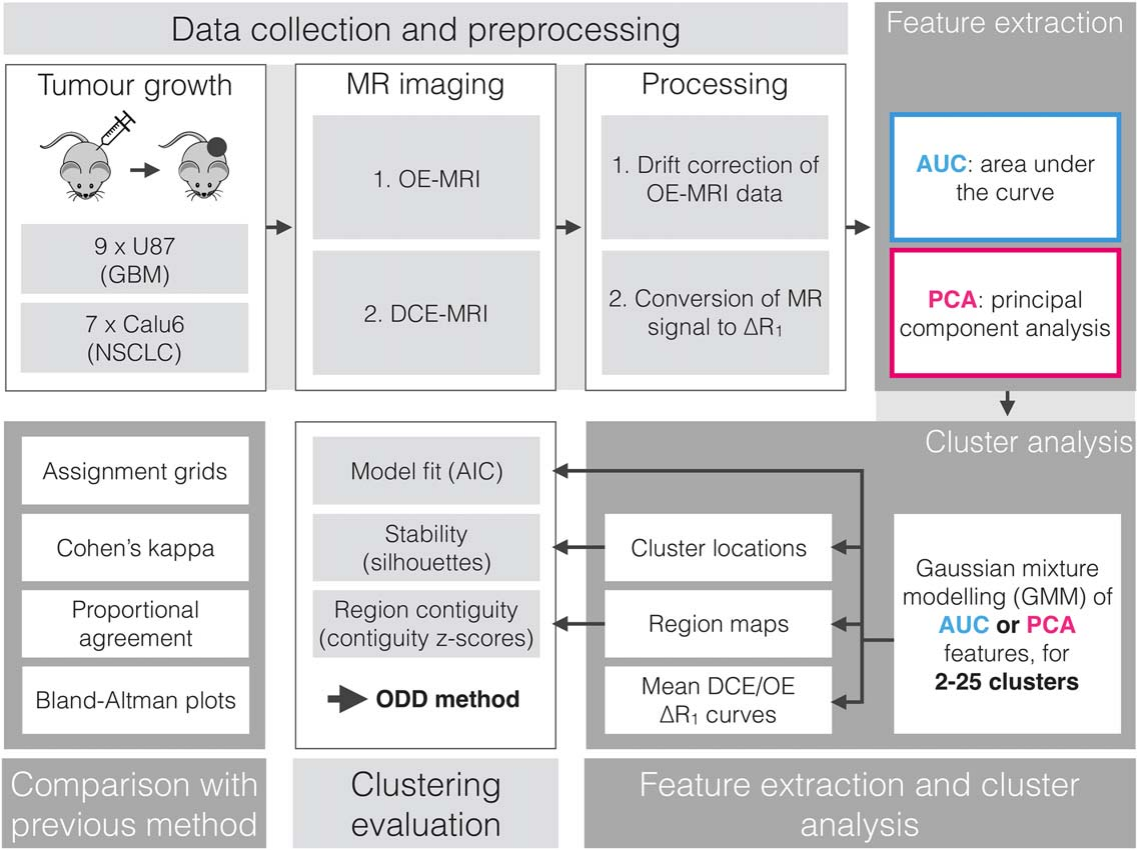
A flowchart depicting the sequence of acquisition, analysis, and evaluations conducted.

All analyses carried out in this work were carried out in MATLAB R2014a (MathWorks, Natick, MA), apart from the calculation of native tissue T_1_ values, which was carried out using in‐house software written in C. Key MATLAB functions are written in italics (e.g., *function.m*). As an overview of the current work, Figure 1 shows graphically the key steps carried out in our analysis stream, organized into the four main sections of work: 1 data collection and preprocessing, 2 feature extraction and cluster analysis, 3 clustering evaluation, and 4 comparison with previous method.

### Data Collection and Preprocessing

Studies were performed in compliance with the National Cancer Research Institute “Guidelines for the welfare and use of animals in cancer research” 43 and with licenses issued under the UK Animals (Scientific Procedures) Act 1986 (PPL 40/3212) following local Ethical Committee review.

Experiments were carried out in two murine xenograft models of human cancer, generated by intra‐dermal injection of cells on the midline lower back of nude mice. Nine U87 glioblastoma multiforme tumor models were propagated by injecting 0.1 ml of cells (5 × 10^6^ cells/ml), and seven Calu6 non‐small cell lung carcinoma tumor models were propagated by injecting 0.1 ml of cells (2 × 10^7^ cells/ml). When tumors reached > 200 mm^3^ by caliper measurement, mice were anaesthetized using 2% isoflurane carried in medical air (21% oxygen). Core temperature was controlled at 37°C while anatomical, OE, and DCE MR imaging was carried out on a 7T Magnex instrument interfaced to a Bruker Advance III console and gradient system using a volume transceiver coil. For each image acquisition, localizer scans were carried out and imaging volumes selected to position tumors centrally within the volume.

#### Anatomical Imaging

A T_2_‐weighted scan was carried out to enable tumor identification and localization. 3D rapid acquisition with relaxation enhancement sequence 44: T_R_ = 2200.00 ms; T_E_ = 32.00 ms; α = 135°; matrix = 96 × 128; field of view = 32 mm × 32 mm; and 16 × 1 mm thick coronal slices.

#### OE‐MRI

A variable flip angle method was carried out to measure tissue baseline T_1_ 45 using 3D modified driven equilibrium Fourier transform sequences 46: T_R_ = 30.00 ms; T_E_ = 1.44 ms; α = 5°, 10°, 20°; five averages; matrix = 64 × 64; field of view = 32 mm × 32 mm; 16 × 1 mm thick coronal slices. This was followed by 42 dynamic acquisitions (acquisition details as above but α = 20° only) at a temporal resolution of one image volume every 28.80 s. The gas supply to the mouse was delivered via a nose cone and switched from air to 100% oxygen at the beginning of the 19th acquisition. The duration of the entire dynamic series was 20 min 10 s.

#### DCE‐MRI

A variable flip angle method was again carried out to measure tissue baseline T_1_ using 3D modified driven equilibrium Fourier transform sequences; T_R_ = 6.02 ms; T_E_ = 1.46 ms; α = 2°, 5°, 10°; five averages; matrix = 64 × 64; field of view = 32 mm × 32 mm; 16 × 1 mm thick coronal slices. This was followed by 96 dynamic acquisitions (acquisition details as above but α = 10° only) at a temporal resolution of one image volume every 5.78 s. Gd‐DOTA was injected into a tail vein (0.25 mmol/kg) at the beginning of the 25th acquisition. The duration of the entire dynamic series was 9 min 15 s.

#### Processing

Native tissue T_1_ (T_10_) values were estimated for both DCE and OE via fitting the spoiled gradient recalled echo signal equation to variable flip angle data 47.

Baseline signal drift was observed in OE data, which was corrected by fitting the spoiled gradient recalled echo signal equation with an empirically determined exponentially time‐varying flip angle. Supporting Figure S1 shows an illustration of the fitted baseline signals.

Dynamic MR signals were then converted from raw signal units to voxel‐wise Δ*R*_1_ (*R*_1_ = 1/T_1_) vectors, by manipulating the spoiled gradient recalled echo signal equation 48 and using T_10_, *S*_0_(*t*), and α(*t*) values for OE, and uncorrected *T*_10_, *S*_0_, and α values for DCE. Supporting Figure S2 shows the effect that drift correction of OE data has on Δ*R*_1_ values.

Regions of interest were drawn around tumor volumes, and only tumor voxels were used in the following analysis. Erratically enhancing voxels that would impede feature calculation were present in most data sets. These existed likely because of movement at a boundary, and perhaps the inclusion of large vessels, in the region of interest. The area‐under‐the‐curve (AUC) of the modulus of each DCE and OE Δ*R*_1_(*t*) curve was calculated, and the voxels with the highest 1% from each imaging technique were removed from the data set to improve the suitability of the data for feature calculation.

### Feature Extraction and Cluster Analysis

Two sets of features were calculated for each tumor voxel: an AUC feature set and a principal component analysis (PCA) feature set.

For the AUC feature set, the area under the first 90 s (post‐Gd) of DCE Δ*R*_1_(*t*) curves and under all post oxygen‐switch time points of OE Δ*R*_1_(*t*) curves was calculated using trapezoidal integration using *cumtrapz.m*.

For the PCA feature set, the DCE and OE Δ*R*_1_(*t*) enhancement curves were concatenated after scaling the OE data so that the mean standard deviation across all voxels for DCE and OE were equal. This created a composite DCE‐OE curve for each voxel. The principal components that describe the directions of greatest variance in DCE‐OE Δ*R*_1_(*t*) enhancement curves, together with voxel weightings for each component, were then calculated using *pca.m*.

Gaussian mixture modeling (GMM) was applied to the two‐dimensional AUC feature set and separately to the four‐dimensional PCA feature set, consisting of the first four principal components, with the number of clusters, *N*_C_, varying from 2 to 25. GMM was implemented using *fitgmdist.m*. Each GMM fit was repeated ten times with randomly initialized cluster locations to avoid local minima. To ensure that clusters in feature space were consistently labeled, every cluster assignment in feature space was reordered so that clusters 1 to *N*_C_ increased from lowest to highest DCE AUC values. For each clustering result (choice of *N*_C_ value and choice of feature set), cluster assignments were transferred into image space, creating a 3D region map for each tumor. Additionally, mean within‐cluster DCE and OE Δ*R*_1_(*t*) curves were calculated.

### Clustering Evaluation

To investigate the suitability of the model fit using GMM clustering, the Akaike information criterion (*AIC*) 49 was used with values derived from the log‐likelihood of GMM fits, for each clustering result.

To assess whether region maps identified connected regions in tumors, a contiguity metric was created. The number of connected regions (3 × 3 × 3 voxel connection kernel) for each tumor region map was calculated for each clustering result using *bwlabeln.m*. The null distribution of this metric was created by bootstrap resampling each region map and calculating the contiguity metric for 100 bootstrap realizations. From these data, contiguity z‐scores were calculated for all tumors for each clustering result.

To evaluate the stability of cluster locations within feature space, both feature sets underwent bootstrap resampling over voxels 100 times. GMM for all *N*_C_ values was then rerun on every bootstrapped feature set and the cluster centers saved. The set of cluster centers from each bootstrap realization were matched to the original cluster centers using the Hungarian algorithm 50, and silhouettes 51 were calculated using *silhouette.m* to quantitatively assess the dispersion of each cluster center for each realization of bootstrap resampling.

The three evaluation metrics (*AIC*, contiguity z‐scores, and stability scores) were compared alongside each other and used to select 1 the feature set and 2 the *N*_C_ value for GMM clustering of this data set. This is the optimized, data‐driven (ODD) method we present.

### Comparison with Previous Method

Alongside the ODD method, the previously published threshold‐based method (TBM) 31 that defined three tumor region classes was also applied to these data. Cluster assignments from ODD methods were compared with the three TBM classes and subsequently concatenated into three similar classes to enable a direct comparison between methods. For each tumor, the proportion of voxels assigned to the same class (φ) and Cohen's kappa (κ) were calculated to quantify the agreement between methods. Bland‐Altman plots of the number of voxels in each of the three classes were also created as a method of assessing methodological agreement.

## RESULTS

### Feature Extraction and Cluster Analysis

**Figure 2:**
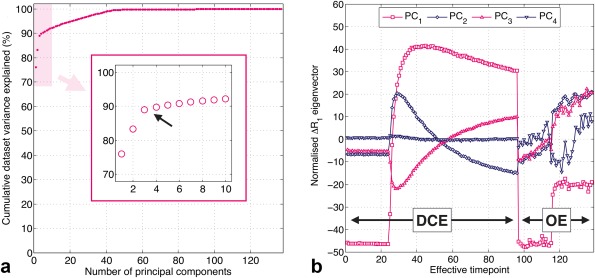
Results from principal component analysis of combined DCE‐OE data (OE Δ*R*_1_(*t*) curves were scaled and concatenated with DCE Δ*R*_1_(*t*) curves). (**a**) Cumulative variance explained with increasing number of principal components, with the arrow and inset highlighting the first shoulder in the curve (∼2–5 components). (**b**) The first four principal components, showing distinct kinetics of combined DCE‐MRI and OE‐MRI enhancement.

Supporting Figure S3 shows T_2_‐weighted images of four representative tumors. Figure 2 shows results from PCA applied to all voxels' DCE‐OE Δ*R*_1_(*t*) time series. Figure 2a shows the cumulative variance explained with increasing number of principal components (PCs). The shoulder at 40–45 PCs most likely reflects the different noise levels within the OE and DCE measurements, as there are 42 OE time points. There is an initial shoulder in the curve at two to five PCs, at which point ∼90% of the variance in the data set is explained (Fig. 2a, inset). The first four PCs were chosen to create the PCA feature set based on the location of this shoulder and visual interpretation of the PCs. Figure 2b shows these first four PCs, with the first part of the curves representing the DCE and the later part the OE Δ*R*_1_(*t*) signal changes. Smooth temporal changes that might be expected because of the administered Gd and oxygen are observed.

**Figure 3:**
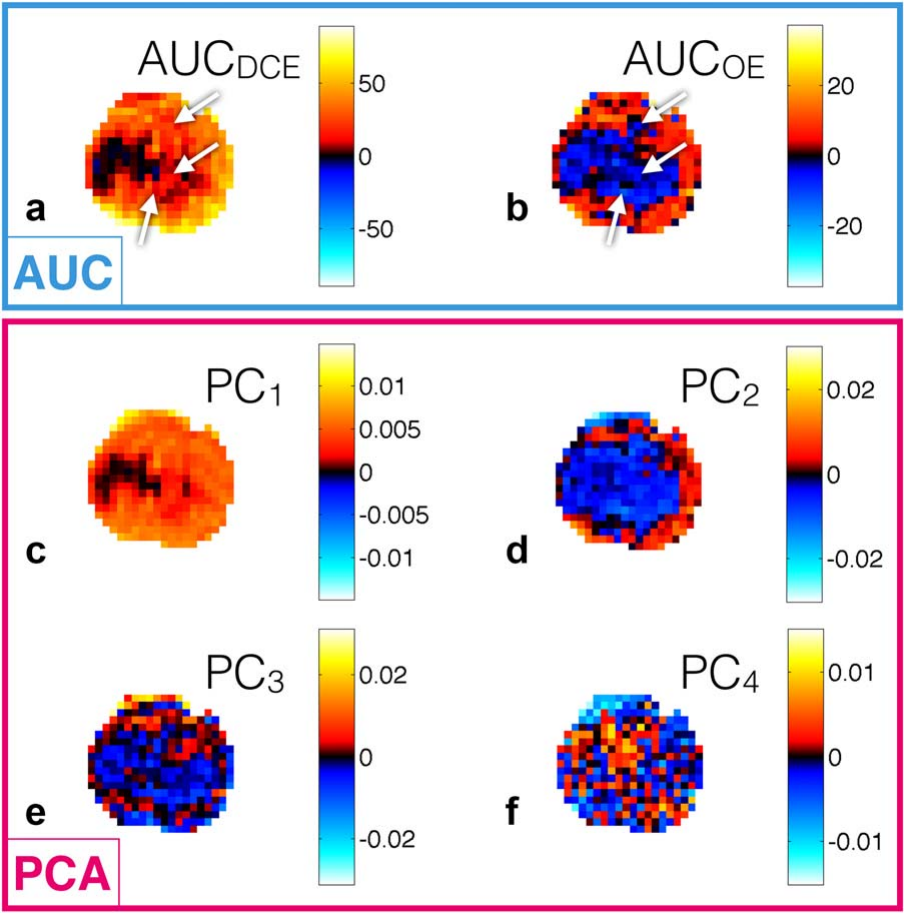
Feature maps for the representative U87 tumor shown in Supporting Figure S3b. (**a** and **b**) Feature maps for the AUC feature set. (**c–f**) Feature maps of PCA weightings for the first four principal components. Arrows highlight regions of AUC_DCE_/AUC_OE_ mismatch. There is structural similarity of (**a**) and (**b**) with (**c**) and (**d**), respectively.

Figure 3 shows feature maps for the central slice of the same tumor in Supporting Figure S3b. AUC maps show a strong rim‐core structure for both features, and regions of high AUC_DCE_ with low or negative AUC_OE_ are observed (see arrows). PC_1_ and PC_2_ feature maps show structures with similarity to those of the AUC_DCE_ and AUC_OE_ features, respectively, whereas PC_3_ and PC_4_ feature maps appear to show spatial structure distinct from that of PC_1_ and PC_2_. Feature maps for all tumors are shown in Supporting Figures S4–S6.

Supporting Figure S7 shows two‐dimensional histograms of the AUC and PCA feature sets.

### Clustering Evaluation

**Figure 4:**
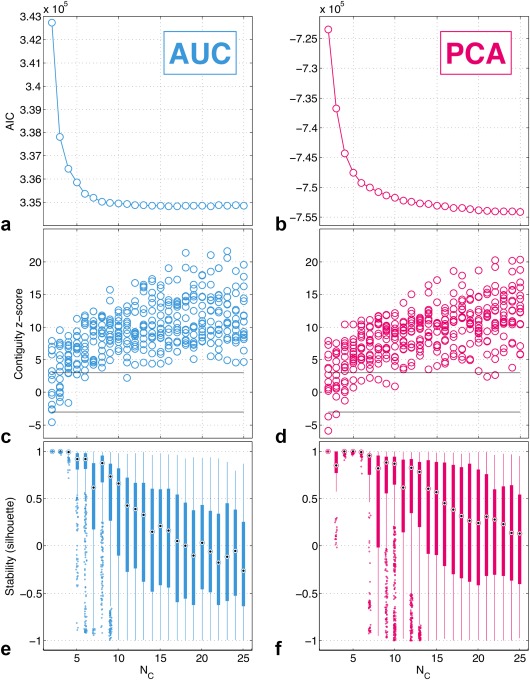
Evaluation metrics from GMM fits of the AUC and PCA feature sets with varying number of clusters (*N*_C_). (**a** and **b**) Akaike information criterion (*AIC*). Neither curve shows a clear minimum, with lower values observed for higher numbers of clusters. (**c** and **d**) Contiguity z‐scores. There are 16 z‐scores for each *N*_C_ value, one for each tumor. Most values lie above 3, indicating statistically significantly greater contiguity in region maps than what would appear because of chance. (**e** and **f**) Stability scores. Each box contains *N*_C_ times 100 cluster centers with a silhouette value calculated for each cluster center. Cluster center locations remain stable (located in similar areas of the feature space with stability scores of close to +1) for up to 4 clusters for AUC, and for up to 6 clusters for PCA.

Figure 4 shows *AIC* values (Figs. 4a, b), contiguity z‐scores (Figs. 4c, d), and stability scores (Figs. 4e, f) from GMM fits with varying *N*_C_ for the AUC and PCA feature sets. For *AIC* values (Figs. 4a, b), neither feature set shows a clear minimum (optimal) value, but both show a steep decrease in *AIC* up to *N*_C_ = 6. After this point, there is a much shallower decrease in *AIC* with increasing *N*_C_, suggesting that only modest improvement in describing the feature set distribution is achieved with more than six clusters, particularly for the AUC feature set. For contiguity z‐scores (Figs. 4c, d), most values are greater than three for all *N*_C_, indicating a probability of <0.3% that the contiguity of regions located in tumor region maps is because of chance. For both feature sets, there is a general trend of increasing contiguity z‐scores in tumors with increasing *N*_C_. For stability scores (Figs. 4e, f), values of + 1 indicate perfect stability of cluster results. Clustering using the AUC feature set results in highly stable cluster centers when *N*_C_ ≤ 4, whereas when using the PCA feature set the cluster centers remain highly stable when *N*_C_ ≤ 6, although less stable cluster centers are observed when *N*_C_ = 3. When *N*_C_ > 6, a steady deterioration in stability is seen for both feature sets.

### ODD Method

*AIC* values suggest that *N*_C_ > 6 gives only marginal increases in GMM fit quality (for both feature sets), and stability scores remain high when *N*_C_ ≤ 6 for the PCA feature set, whereas for the AUC feature set stability only remains high when *N*_C_ ≤ 4. The optimized feature set and number of clusters for this data set was therefore determined to be clustering the PCA feature set with *N*_C_ = 6.

**Figure 5:**
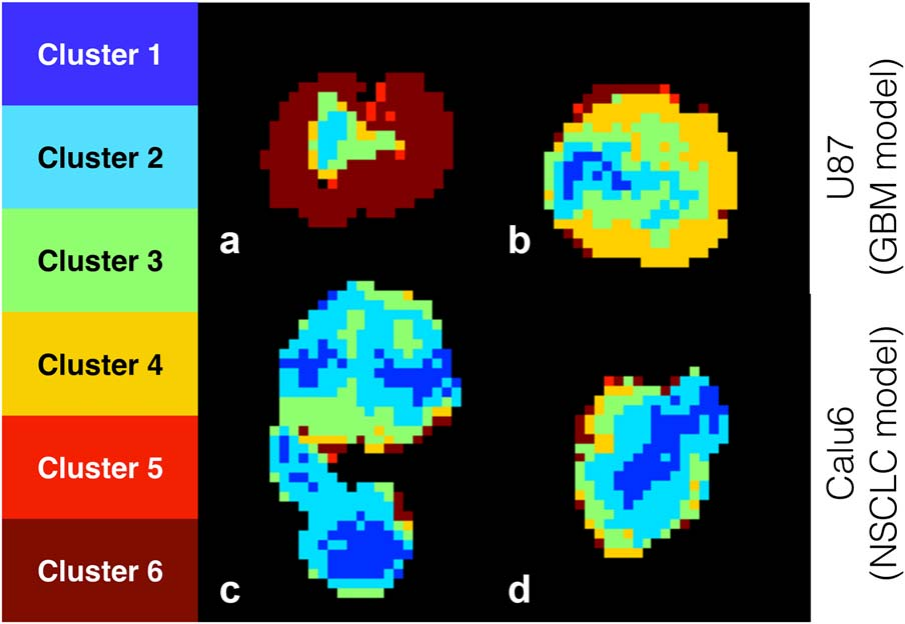
Results from the ODD method. Tumor region maps for the two representative U87 tumors and two representative Calu6 tumors shown in Supporting Figure S3. Largely contiguous regions are located, with rim‐core structures present in most tumors.

**Figure 6:**
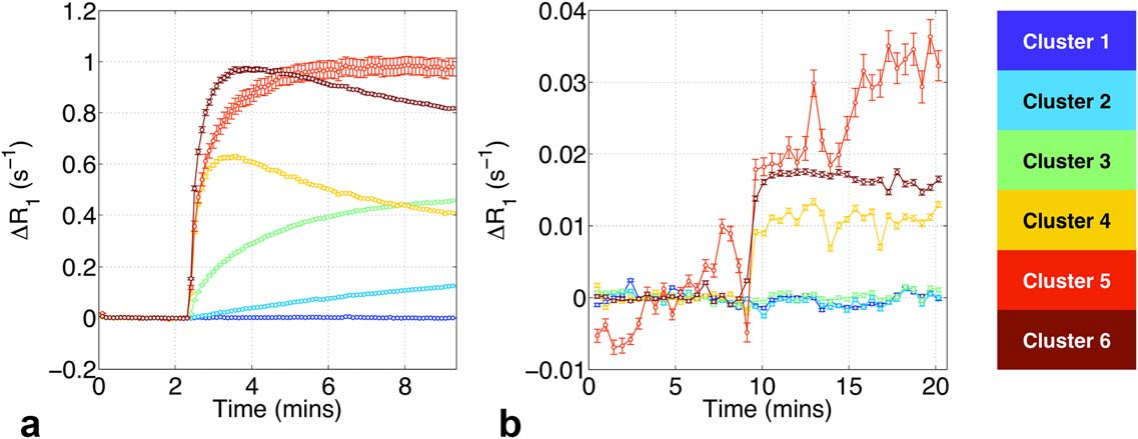
Results from the ODD method. Mean within‐cluster Δ*R*_1_(*t*) enhancement curves for DCE‐MRI (**a**) and OE‐MRI (**b**). Error bars show standard error of the mean. Curves show distinct, intuitive kinetics of enhancement, with lack of overlap in the post‐contrast regions. Clusters 2 and 3 (light blue and green) show DCE‐MRI enhancement with no OE‐MRI enhancement, possibly linked with hypoxia.

Results from the ODD method are shown in Figures 5 and 6 and Supporting Figures S8 and S9.

Figure 5 shows tumor region maps from the ODD method for the same four representative tumors shown in Supporting Figure S3. Contiguous regions are located, with the exception of a few voxels on the tumor periphery, and tumors show a rough rim‐core structure. Heterogeneous structures are located in approximately half of the tumors, exemplified by Figures 5b, c. Region maps have different cluster compositions depending on tumor line: voxels in clusters 1 and 2 predominantly belong to Calu6 tumors (694 U87 voxels, 8050 Calu6 voxels, 1:11.6 ratio) whereas voxels in clusters 4 and 6 are predominantly in U87 tumors (7180 U87 voxels, 1467 Calu6 voxels, 4.89:1 ratio).

Figure 6 shows mean within‐cluster DCE and OE Δ*R*_1_(*t*) enhancement curves defined using the ODD method, with error bars of the standard error of the mean plotted. Curves show distinct kinetics of enhancement following Gd administration (Fig. 6a) and the oxygen‐switch (Fig. 6b). Cluster 1 enhances in neither DCE nor OE, clusters 2 and 3 enhance only modestly in DCE, and clusters 4–6 enhance strongly in both DCE and OE. Regions with these characteristics have been previously shown to correspond to necrotic, hypoxic, and well‐perfused/well‐oxygenated tumor tissue, respectively, in multiple xenograft models 31. For our work, correlative histology was not available, and caution must be taken in assigning biological interpretations to clusters.

### Comparison with Previous Method

Supporting Figure S10 shows assignment grids showing how tumor voxels are classified using the ODD method and TBM. Inspection of mean enhancement curves provided justification for concatenating clusters 2 and 3 into a single group, and 4, 5, and 6 into another group. This enabled further comparison of ODD methods with TBM. Supporting Figure S11 shows a side‐by‐side comparison of tumor region maps created using both methods. The majority of tumors show a strong visual concordance between methods. Supporting Figure S12 shows proportional agreement (φ) and Cohen's kappa (κ) statistics, quantifying the agreement between methods of categorizing tumor voxels. Looking at the group values in Supporting Figure S12, both statistics show good agreement between methods: φ = 0.75 and κ = 0.61. The low values in tumors 3, 5, 14, and 15 correspond to cases where the majority of voxels are classified into a single class using one of the methods and so κ gives artificially low values. Supporting Figure S13 shows Bland‐Altman plots for the number of voxels in each of the three categories determined by both methods.

## DISCUSSION

The current work presents a thorough investigation into the application of data‐led clustering methods to MRI data designed to identify putatively hypoxic tumor tissue, putting forward a combination of evaluation metrics that enable objective methodological optimization. Our results agree well with the previously published work 31, 32, however we demonstrate the ability of the data to robustly support more than the three tissue classes previously located, indicating that there may be further information to exploit in the combination of DCE and OE data if methods such as those we present here are adopted.

Soft clustering methods (where clusters may overlap) generally perform more robustly than hard clustering methods (where clusters cannot overlap) under high levels of noise and when data distributions are smoothly varying. GMM was chosen as it is a well‐known soft clustering technique that gives a probabilistic assignment of each voxel to each cluster, though the most probable cluster for each voxel was used as a hard classification. In this way, the final output is clear‐cut and can be easily associated with a physiological interpretation. However, this can lead to classification errors in borderline data lying between two clusters, but maintaining high cluster assignment robustness is intrinsic to the ODD method and should mitigate errors of this sort. The probabilistic cluster assignment from GMM could be maintained or an alternative soft clustering method (e.g., fuzzy c‐means) used, which would avoid these errors. This might provide insight into the overlap, or lack thereof, of biological habitats in tumors, or at least allow the identification of regions with poorly categorized features.

It is far from trivial to select the most appropriate clustering method, lending importance to our combination of evaluation metrics. Contiguity z‐scores (Figs. 4c, d) inform us that transferring cluster assignments into image space gives genuine structure, as opposed to the random categorization of voxels. This suggests that located tumor regions are based on biology, but does not provide a strong basis for selecting the most appropriate feature set or number of clusters. The absence of clear minima in *AIC* values (Figs. 4a, b) is likely because of the lack of distinct voxel groupings in feature space. However, the shoulders in *AIC* curves suggest that we do need at least six clusters to adequately fit the data. The stability analysis (Figs. 4e, f) provides an objective measure of the number of clusters that can be reliably supported by these data. From these metrics, running GMM clustering on the AUC feature set with four or fewer clusters and the PCA feature set with six or fewer clusters appeared to be optimal. Based on this, we selected the PCA feature set with the most clusters that the data could robustly support (and therefore more tissue classes), potentially providing more insight into the tumor microenvironment and its heterogeneity. PCA also has the advantage of requiring fewer a priori assumptions in the analysis. We desire a high number of clusters to identify features of potential importance and adequately characterize tumor heterogeneity, while simultaneously desiring high repeatability and reliability of our results, which typically deteriorates as the number of clusters increases. If greater than the optimum number of clusters is used, the clustering is increasingly subject to random errors in the data, and if fewer are used, the clustering fails to account for potentially important information contained in the data. The combination of evaluation methods used in this study provides an objective measure of where to place this trade‐off—selecting the highest number of stable clusters the data can support and ensuring that significant regional contiguity exists in tumor region maps.

A potential validation of this work that was not carried out was a comparison of region maps with histologically stained tumor sections. The tumors investigated were part of a separate longitudinal radiotherapy study; all imaging data in the current study is from the baseline, pre‐radiotherapy visit. Histological staining was acquired, but treatment effects and significant tumor growth before excision (up to 14 days later) prevent any valid comparisons being made with the imaging data used in the current study. However, TBM has itself been histologically validated (pimonidazole staining correlating strongly with perfused Oxy‐R fraction 31), and we demonstrate strong agreement between the ODD method and TBM, cross‐validating both techniques. ODD results were necessarily simplified (six classes combined into three) to enable comparisons with TBM. A significant strength of this work therefore lies in the finer distinction the ODD method makes between tissue classes, but, in the absence of ground truth histological data, we are unable to definitively interpret the biological relevance of this distinction.

## CONCLUSIONS

The development of a non‐invasive, objective, and robust tool for characterizing the tumor microenvironment is necessitated by the heterogeneous nature of tumor tissue and the push toward personalized medicine. Here, we present a data‐led methodology for classifying multi‐spectral tumor imaging data, alongside methods for optimizing parameter choices. We apply our methods to a cohort of DCE‐MRI and OE‐MRI preclinical tumor data, demonstrating the ability of our methods to extract physiologically distinct regions within tumors. Oxygenation and perfusion related characteristics are successfully spatially mapped in the tumors studied, with varying degrees of intra‐tumoral heterogeneity across the cohort. We show that our methodology agrees well with a previously published method for locating hypoxia in tumors, itself validated with correlative histology, and our methods identify a greater number of distinct tumor regions than the previous method. Further work is required to ascertain the biological significance of this result, and future work will include the application of our methods in human tumors, assessing the potential of combined DCE/OE‐MRI clustering in a clinical setting.

## Supporting information


**Fig. S1**. Mean OE‐MRI signal values from each tumor (blue circles), with the fit of an exponentially time‐varying baseline to pre‐oxygen enhancement time points shown, and extrapolated to the post contrast time points (pink line).
**Fig. S2**. Mean OE‐MRI ΔR_1_(t) values for each tumor, calculated without any form of drift correction (blue circles) and with our drift correction (pink circles).
**Fig. S3**. Representative T_2_‐weighted images of central slices through two U87 tumors (**a** and **b**) and two Calu6 tumors (**c** and **d**), demonstrating tumor anatomy and acquisition field‐of‐view. (**a**) An irregularly shaped tumor with a hyper‐intense region, possibly corresponding to edema. (**c**) A tumor with an even more irregular, bi‐lobular shape, but with comparatively homogeneous signal intensities. (**b**) A largely homogeneous tumor with a circular cross‐section. (**d**) A tumor with a similar level of homogeneity but with an elliptical cross‐section.
**Fig. S4**. AUC_DCE_ and PC1 (first principal component) feature maps for a central slice through all 16 tumors. Note the similarity in structure in most tumors between AUC_DCE_ and PC1.
**Fig. S5**. AUC_OE_ and PC2 (second principal component) feature maps for a central slice through all 16 tumors. Note the similarity in structure in most tumors between AUC_OE_ and PC2.
**Fig. S6**. PC3 and PC4 (third and fourth principal component) feature maps for a central slice through all 16 tumors.
**Fig. S7**. Two‐dimensional histograms of the AUC and PCA feature sets alongside Spearman's ρ values, with the four‐dimensional PCA feature set split into its two‐dimensional projections. The AUC feature set shows moderate correlation between the inputs, whereas the PCA feature set shows no strong correlation, indicating good separation of information between the four components. Neither feature set shows clear, distinct separations between voxel groupings, but we observe smooth changes in density of the feature space distributions. All plots show a dense occupation of feature space around the origin (yellow histogram bins).
**Fig. S8**. Results from the ODD method. Cluster assignments in feature space. Plots show two‐dimensional projections of the PCA feature set, with color‐coded cluster assignments to voxels. Clusters 1 and 2 (dark and light blue) show a large overlap, which describes the dense region around the origin in feature space, with clusters 3, 4, and 6 (green, yellow, and brown) showing much less overlap and characterizing the less dense regions of feature space. Cluster 5 (red) represents a large, diffuse Gaussian distribution of voxels that do not appear to belong to any of the other clusters.
**Fig. S9**. Results from the ODD method. Tumor region maps for central slices of all tumors, with color‐coded cluster assignments to voxels. Rough rim‐core structures are present in most tumors.
**Fig. S10**. Assignments grids showing how the previously published TBM and our ODD method compare at assigning voxels to categories (top row). After concatenating the six categories from ODD into three classes, the bottom row shows assignment grids of TBM versus ODD (cat), with proportional agreement (φ) and Cohen's kappa (κ) statistics calculated.
**Fig. S11**. Side‐by‐side comparison of region maps from the previously published TBM and from the optimized, data‐driven method concatenated into three classes (ODD [cat]).
**Fig. S12**. Proportional agreement (φ) and Cohen's kappa (κ), calculated to rate agreement between the previously published, TBM and the ODD method. Values were calculated for individual tumors, the minimum, median, and max value highlighted, and group statistics were calculated for each tumor line and for the whole cohort.
**Fig. S13**. Bland‐Altman plots for the number of voxels in each of the three categories determined using the previously published TBM and using the ODD method. For categories 1, 2, and 3, the bias in voxel categorization (ODD − TBM) is −19.7, + 115.6, and −95.9 voxels, corresponding to (−)1.4%, 8.1%, and (−)6.8% of the mean tumor size (1419.2 voxels).
