# Supplementary material for: Data‐driven mapping of hypoxia‐related tumor heterogeneity using DCE‐MRI and OE‐MRI
Source: Magn Reson Med. 2017 Aug 30;79(4):2236–45. doi: 10.1002/mrm.26860 (PMC5836865; doi:10.1002/mrm.26860)
Supplement: Supplementary file 1 — Fig. S1. Mean OE‐MRI signal values from each tumor (blue circles), with the fit of an exponentially time‐varying baseline to pre‐oxygen enhancement time points shown, and extrapolated to the post contrast time points (pink line). Fig. S2. Mean OE‐MRI ΔR1(t) values for each tumor, calculated without any form of drift correction (blue circles) and with our drift correction (pink circles). Fig. S3. Representative T2‐weighted images of central slices through two U87 tumors (a and b) and two Calu6 tumors (c and d), demonstrating tumor anatomy and acquisition field‐of‐view. (a) An irregularly shaped tumor with a hyper‐intense region, possibly corresponding to edema. (c) A tumor with an even more irregular, bi‐lobular shape, but with comparatively homogeneous signal intensities. (b) A largely homogeneous tumor with a circular cross‐section. (d) A tumor with a similar level of homogeneity but with an elliptical cross‐section. Fig. S4. AUCDCE and PC1 (first principal component) feature maps for a central slice through all 16 tumors. Note the similarity in structure in most tumors between AUCDCE and PC1. Fig. S5. AUCOE and PC2 (second principal component) feature maps for a central slice through all 16 tumors. Note the similarity in structure in most tumors between AUCOE and PC2. Fig. S6. PC3 and PC4 (third and fourth principal component) feature maps for a central slice through all 16 tumors. Fig. S7. Two‐dimensional histograms of the AUC and PCA feature sets alongside Spearman's ρ values, with the four‐dimensional PCA feature set split into its two‐dimensional projections. The AUC feature set shows moderate correlation between the inputs, whereas the PCA feature set shows no strong correlation, indicating good separation of information between the four components. Neither feature set shows clear, distinct separations between voxel groupings, but we observe smooth changes in density of the feature space distributions. All plots show a dense occupation of feature space around [file MRM-79-2236-s001.pdf]

SUPPORTING INFORMATION

A) Drift correction of OE-MRI data

Method

Baseline signal drift was observed in the OE-MRI data, which was corrected by fitting the SPGR signal equation with an empirically-determined exponentially time-varying flip angle, $\alpha(t)$,

$$\alpha(t) = (\alpha_0 - \alpha_f)\exp(-\gamma t) + \alpha_f, \quad \text{(Eq. 1)}$$

designed to account for B_1 drift, to the pre-oxygen switch signal values, where α_0 and α_f are the initial and asymptotic final flip angles , respectively, and γ is the rate constant of the exponential.

Fitting was performed on the first 18 time points using *fminsearch.m*, with α_0 fixed to the value determined by the automatic scanner calibration under the assumption that the flip angle set by the scanner is accurate prior to the dynamic series. The fitting used separate values of α_f for each tumor, under the assumption that the overall degree of drift in flip angle is dependent on the scanner state at the time of scanning, with a common value of γ across the whole tumor cohort based on observation of the data and on the assumption that any change in flip angle is a machine characteristic.

A signal reflecting the change in non-enhanced (baseline) signal over time, $S(t)$,

$$S(x,t) = \frac{M_0(x)\sin(\alpha(t))\left(1-\exp\left(\frac{-T_R}{T_1(x)}\right)\right)}{1-\cos(\alpha(t))\exp\left(\frac{-T_R}{T_1(x)}\right)}, \quad \text{(Eq. 2)}$$

was created, which extrapolates this fit to later time points, where M_0 is the relative proton spin density, T_1 is the longitudinal relaxation time, and T_R is

the repetition time prescribed for the sequence. Note that M_0 and T_1 are spatially variant.

Results

An illustration of the OE-MRI signal drift characterisation and correction is shown in supporting Figs. S1 and S2. Fig. S1 shows the MR signal in each tumor with fitted $S_0(t)$ curves overlaid, which appear to appropriately characterise the pre oxygen-switch time points. Fig. S2 shows corresponding $\Delta R_1(t)$ values calculated using the fitted $S_0(t)$, and also $\Delta R_1(t)$ values calculated with no drift correction, illustrating the impact correction has. Corrected $\Delta R_1(t)$ values appear physiologically plausible and curves appear drift-free, with the OE-MRI response approximating a step function of different magnitudes.

Discussion

Drift correction of OE-MRI data was necessary as the pre oxygen-switch signal drift was of the same order of magnitude as the signal enhancement post oxygen-switch (Fig. S1), therefore large errors could be introduced into calculated $\Delta R_1(t)$ values and the interpretation of oxygen enhancement if the drift was not correctly accounted for. The choice of an exponential form for $\alpha(t)$ was made after visual inspection of pre-contrast MR signal values in the tumors (Fig. S1) and with the hypothesis that some global effect, such as heating in the MRI hardware leading to flip angle variation, was the cause of the signal drift. The consistency of the drift exponential across tumors is consistent with this hypothesis. Nevertheless, other explanations for the observed drift cannot be excluded. The methods used appear to effectively remove observed drift, and do so consistently across all tumors (Fig. S2), suggesting that the methods proposed are both necessary and adequate.

B) Supporting figures

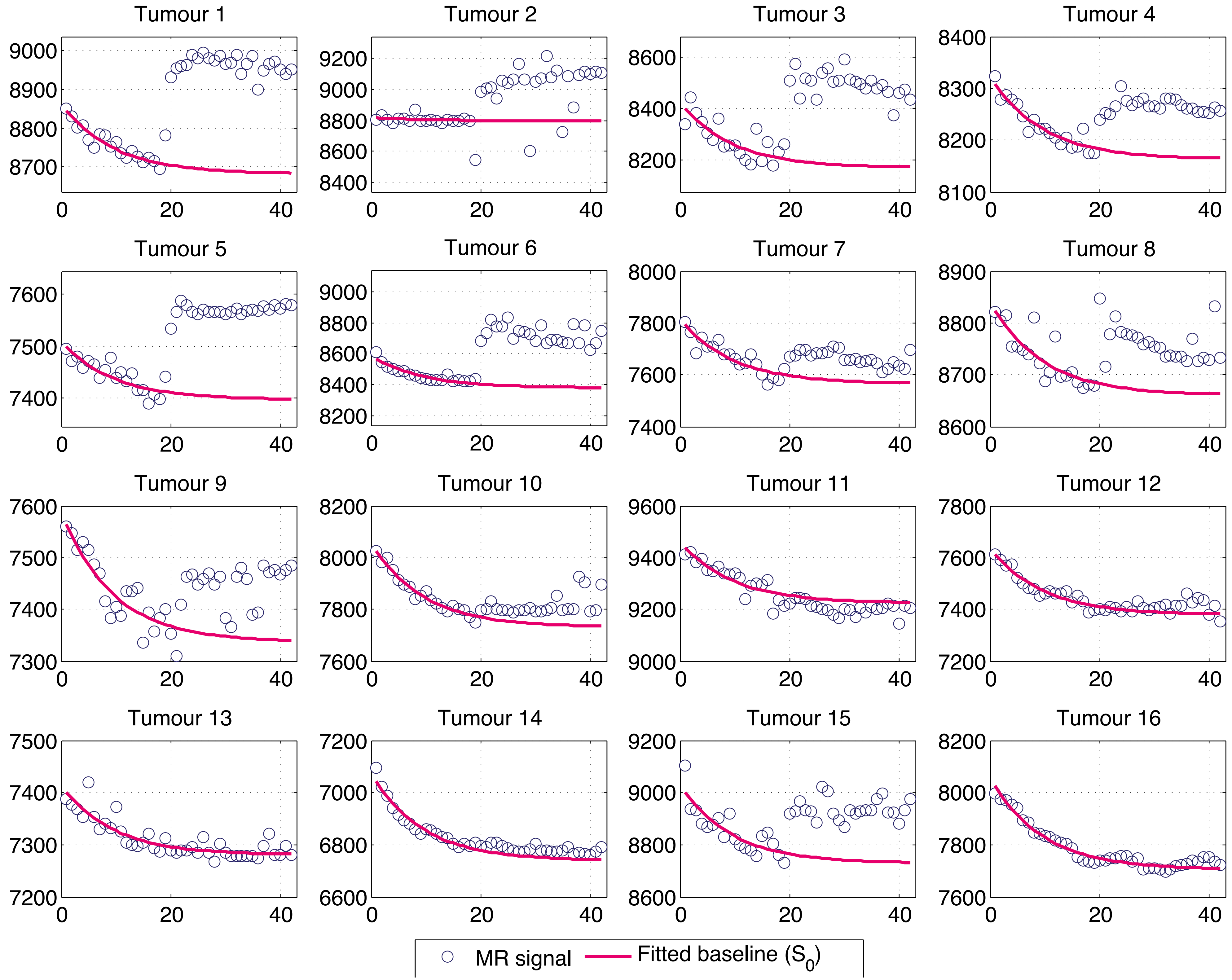

Fig. S1: Mean OE-MRI signal values from each tumor (blue circles), with the fit of an exponentially time-varying baseline to pre-oxygen enhancement time points shown, and extrapolated to the post contrast time points (pink line).

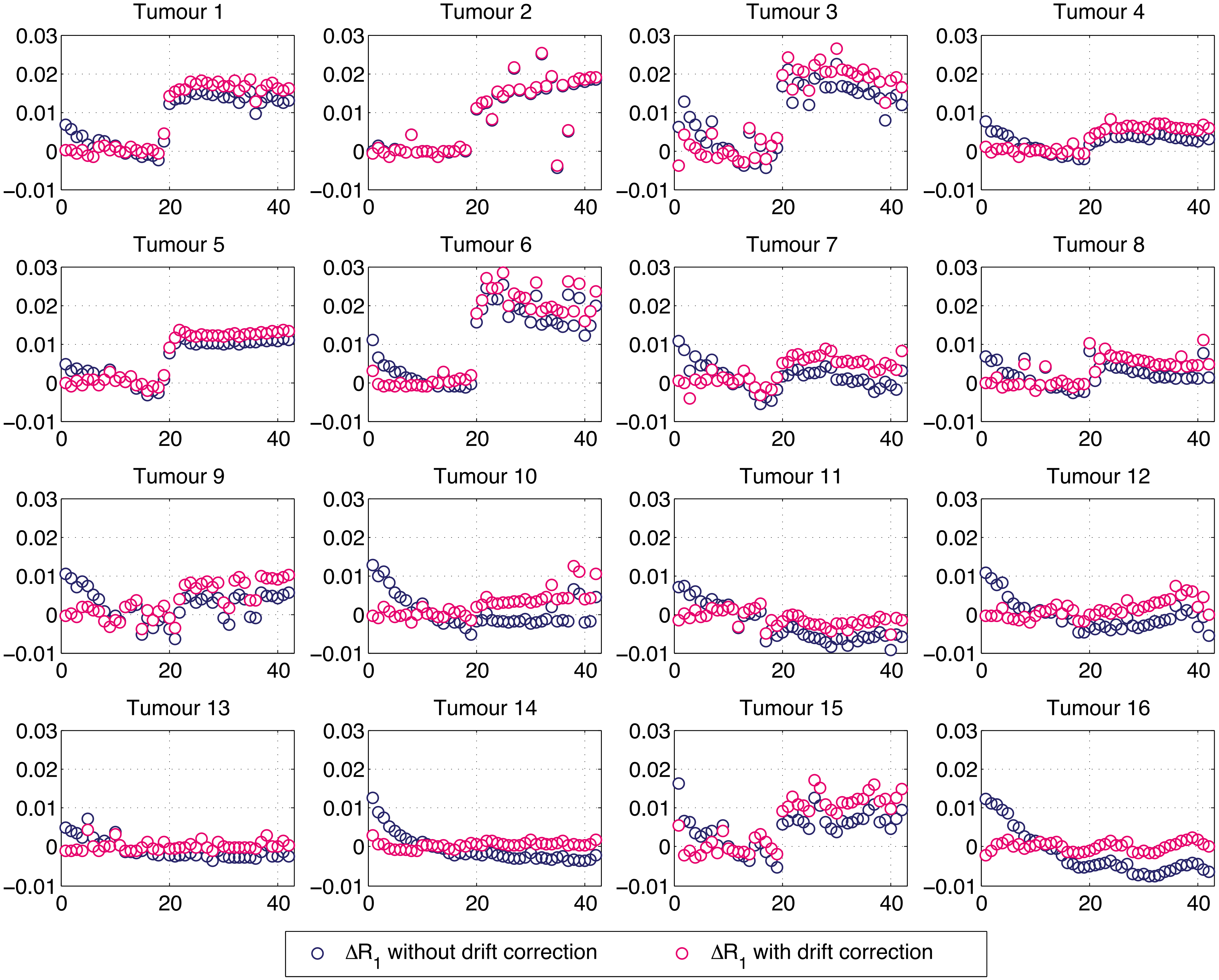

Fig. S2: Mean OE-MRI ΔR1(t) values for each tumor, calculated without any form of drift correction (blue circles) and with our drift correction (pink circles).

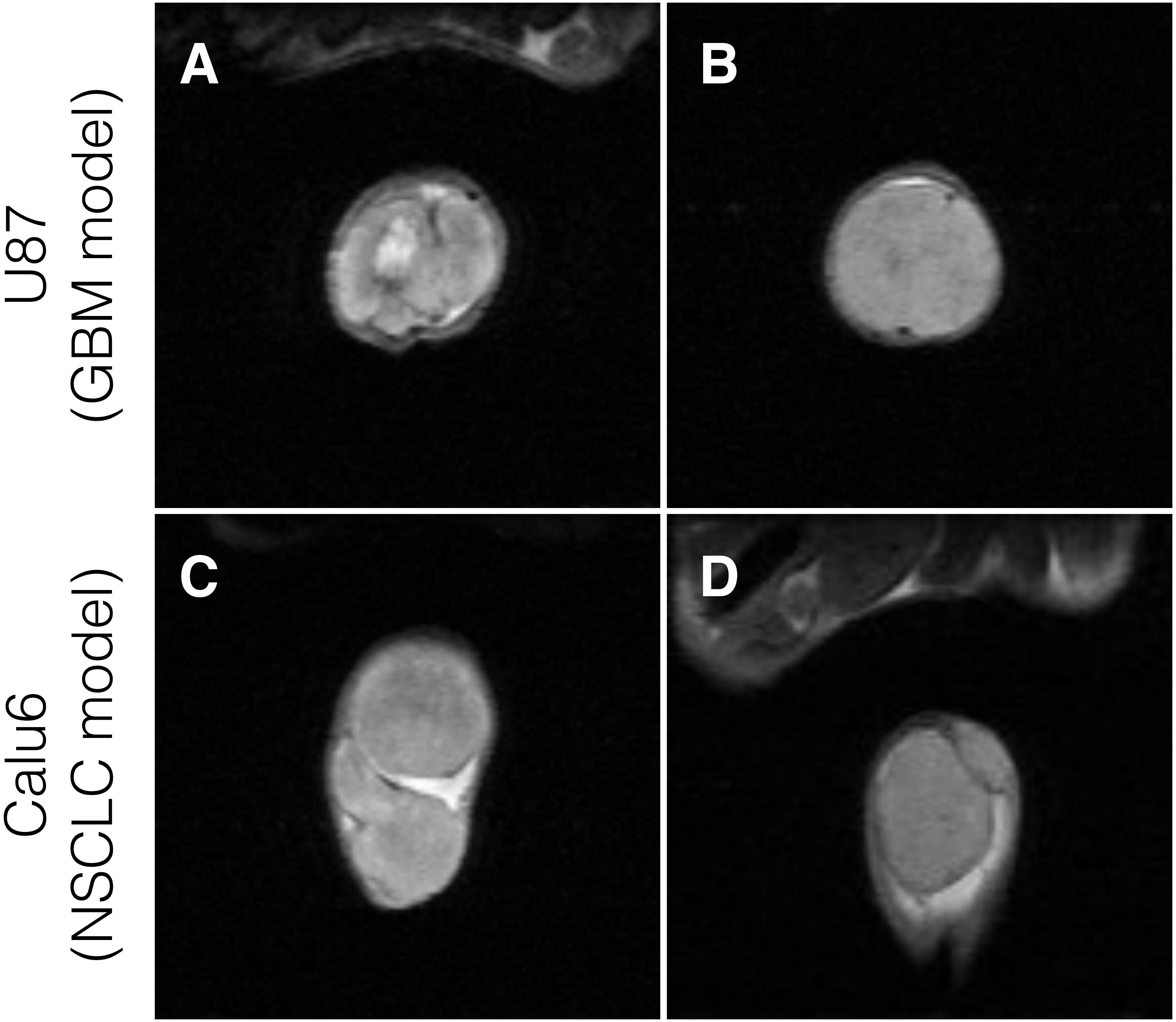

Fig. S3: Representative T2-weighted images of central slices through two U87 tumors (A and B) and two Calu6 tumors (C and D), demonstrating tumor anatomy and acquisition field-of-view. A shows an irregularly shaped tumor with a hyperintense region, possibly corresponding to edema, whilst C shows a tumor with an even more irregular, bi-lobular shape, but with comparatively homogeneous signal intensities. B shows a largely homogeneous tumor with a circular cross-section, and D shows a tumor with a similar level of homogeneity but with an elliptical cross-section.

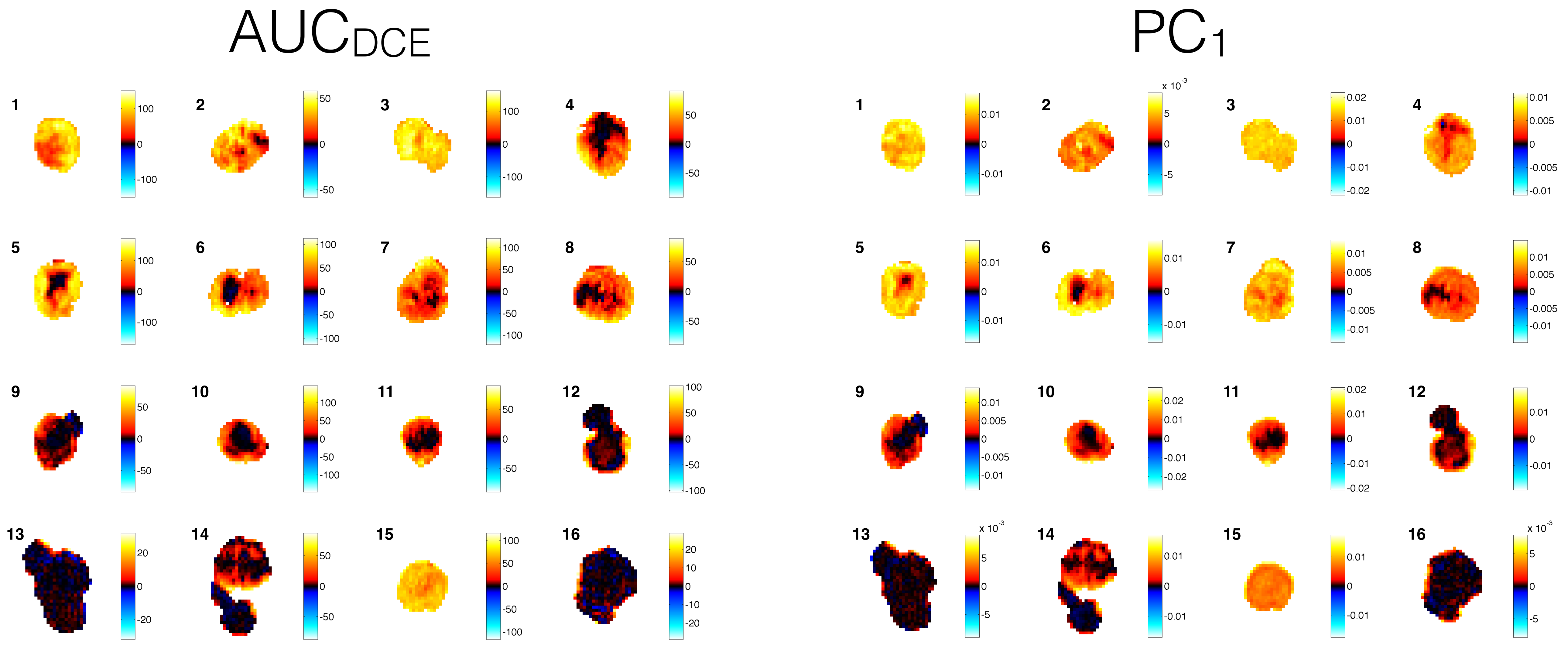

Fig. S4: AUCDCE and PC1 (first principal component) feature maps for a central slice through all 16 tumors. Note the similarity in structure in most tumors between AUCDCE and PC1.

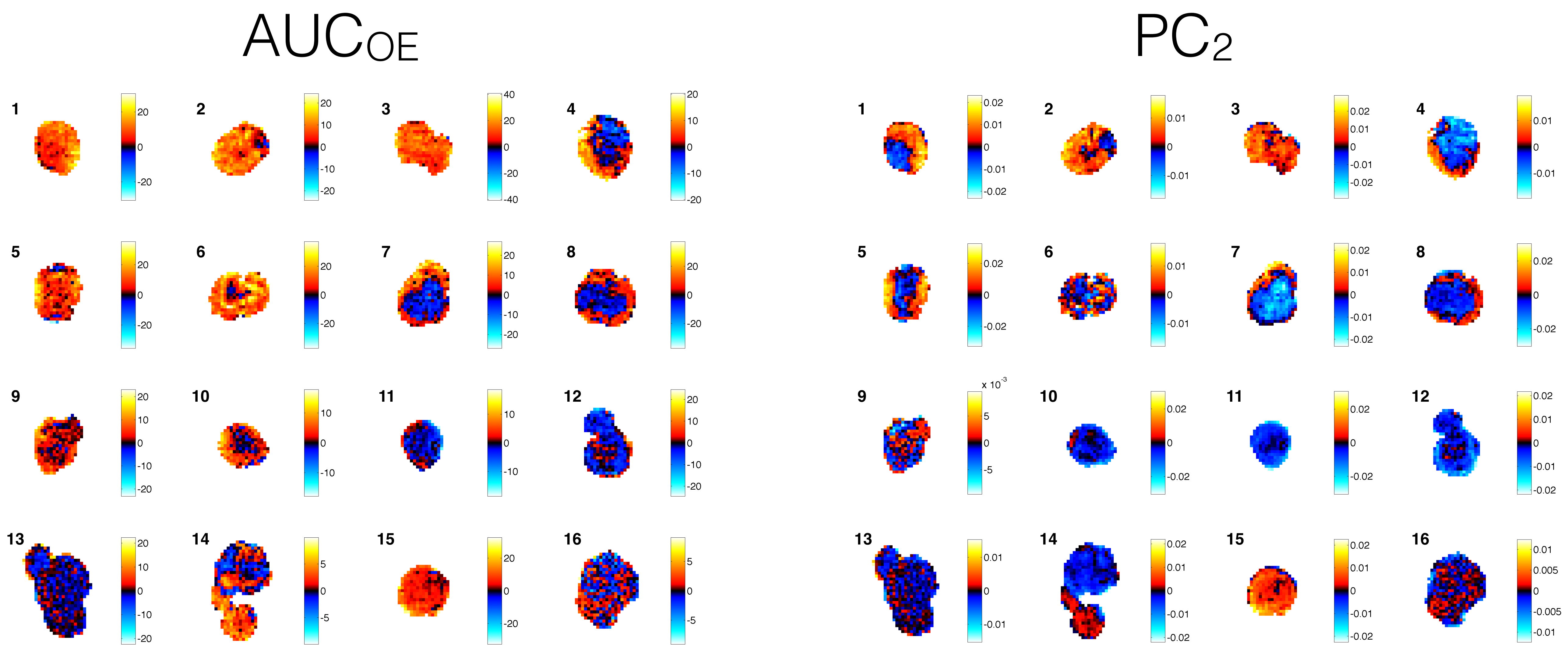

Fig. S5: AUCOE and PC2 (second principal component) feature maps for a central slice through all 16 tumors. Note the similarity in structure in most tumors between AUCOE and PC2.

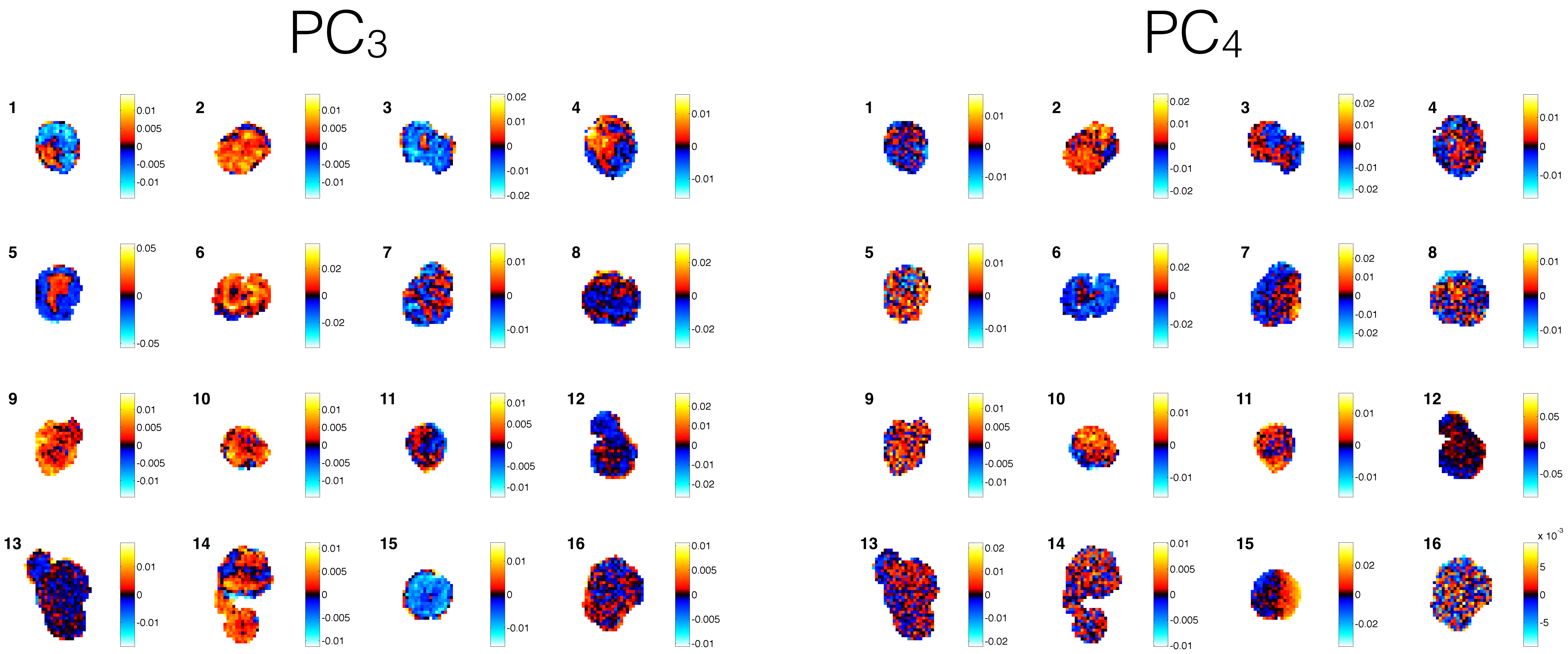

Fig. S6: PC3 and PC4 (third and fourth principal component) feature maps for a central slice through all 16 tumors.

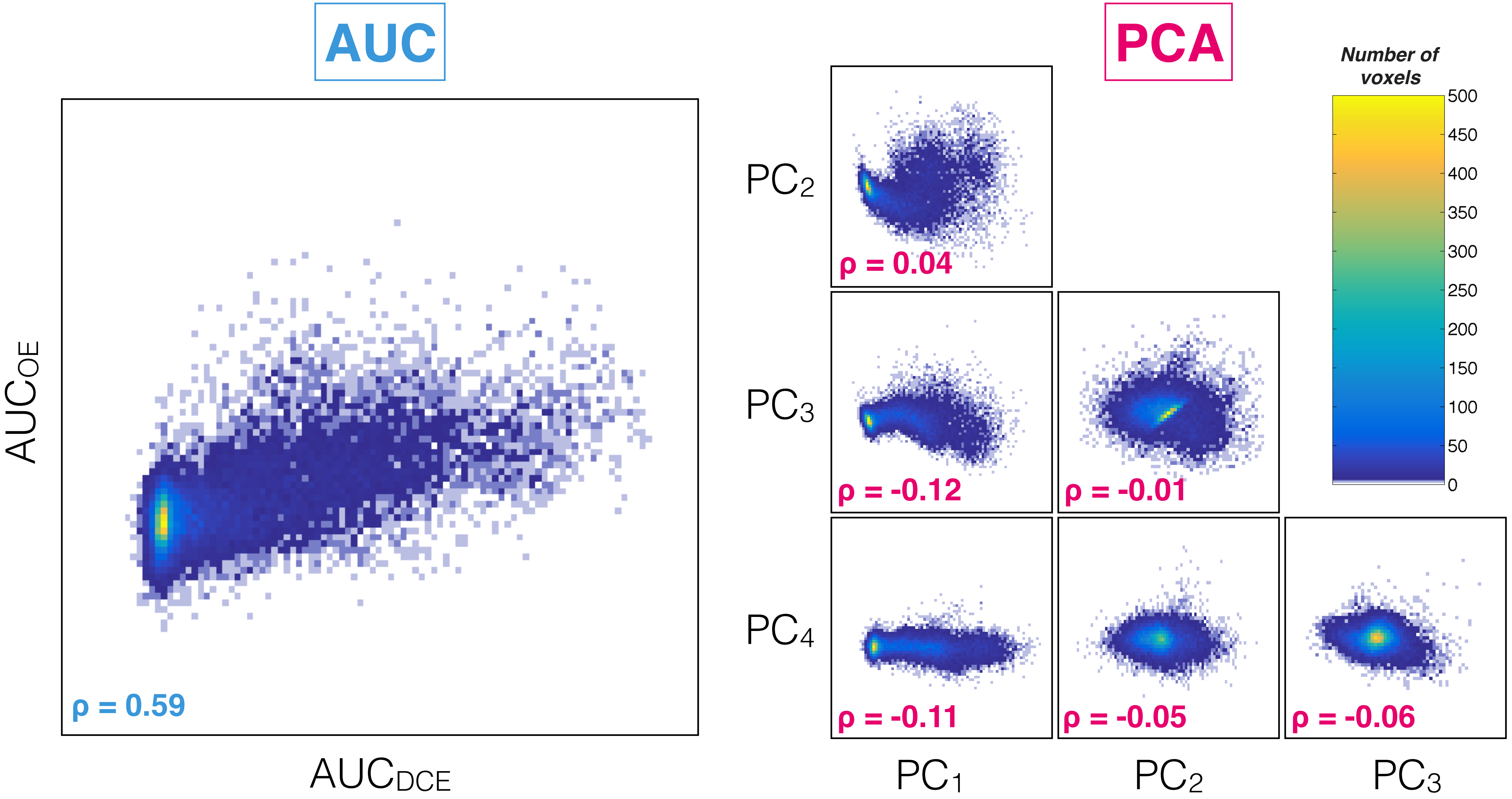

Fig. S7: Two-dimensional histograms of the AUC and PCA feature sets alongside Spearman's ρ values, with the four-dimensional PCA feature set split into its two-dimensional projections. The AUC feature set shows moderate correlation between the inputs, whereas the PCA feature set shows no strong correlation, indicating good separation of information between the four components. Neither feature set shows clear, distinct separations between voxel groupings, but we observe smooth changes in density of the feature space distributions. All plots show a dense occupation of feature space around the origin (yellow histogram bins).

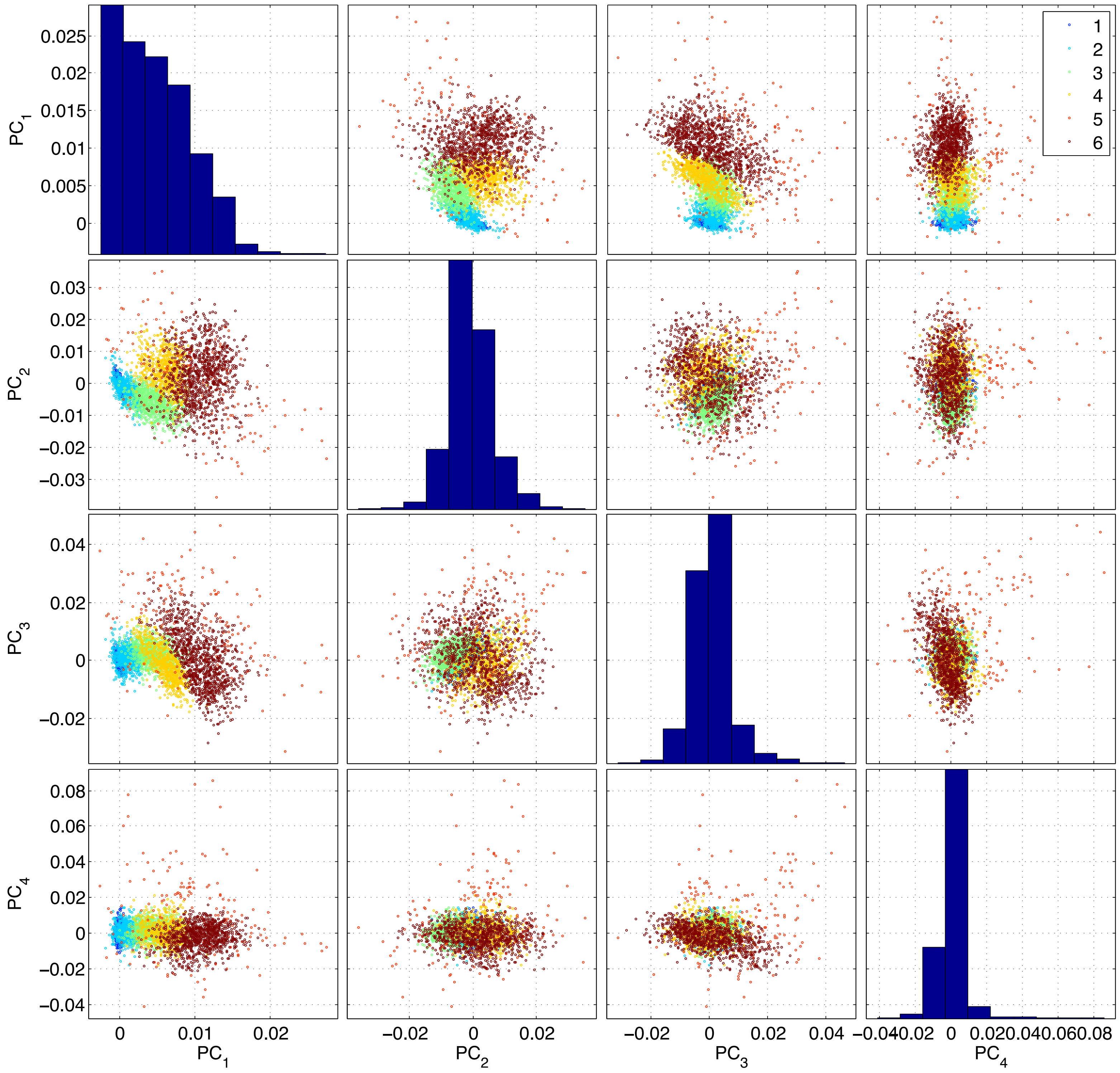

Fig. S8: Results from the optimized, data-driven (ODD) method: cluster assignments in feature space. Plots show two-dimensional projections of the PCA feature set, with colour coded cluster assignments to voxels. Clusters 1 and 2 (dark and light blue) show a large overlap, which describes the dense region around the origin in feature space, with clusters 3, 4, and 6 (green, yellow, and brown) showing much less overlap and characterising the less dense regions of feature space. Cluster 5 (red) represents a large, diffuse Gaussian distribution of voxels that do not appear to belong to any of the other clusters.

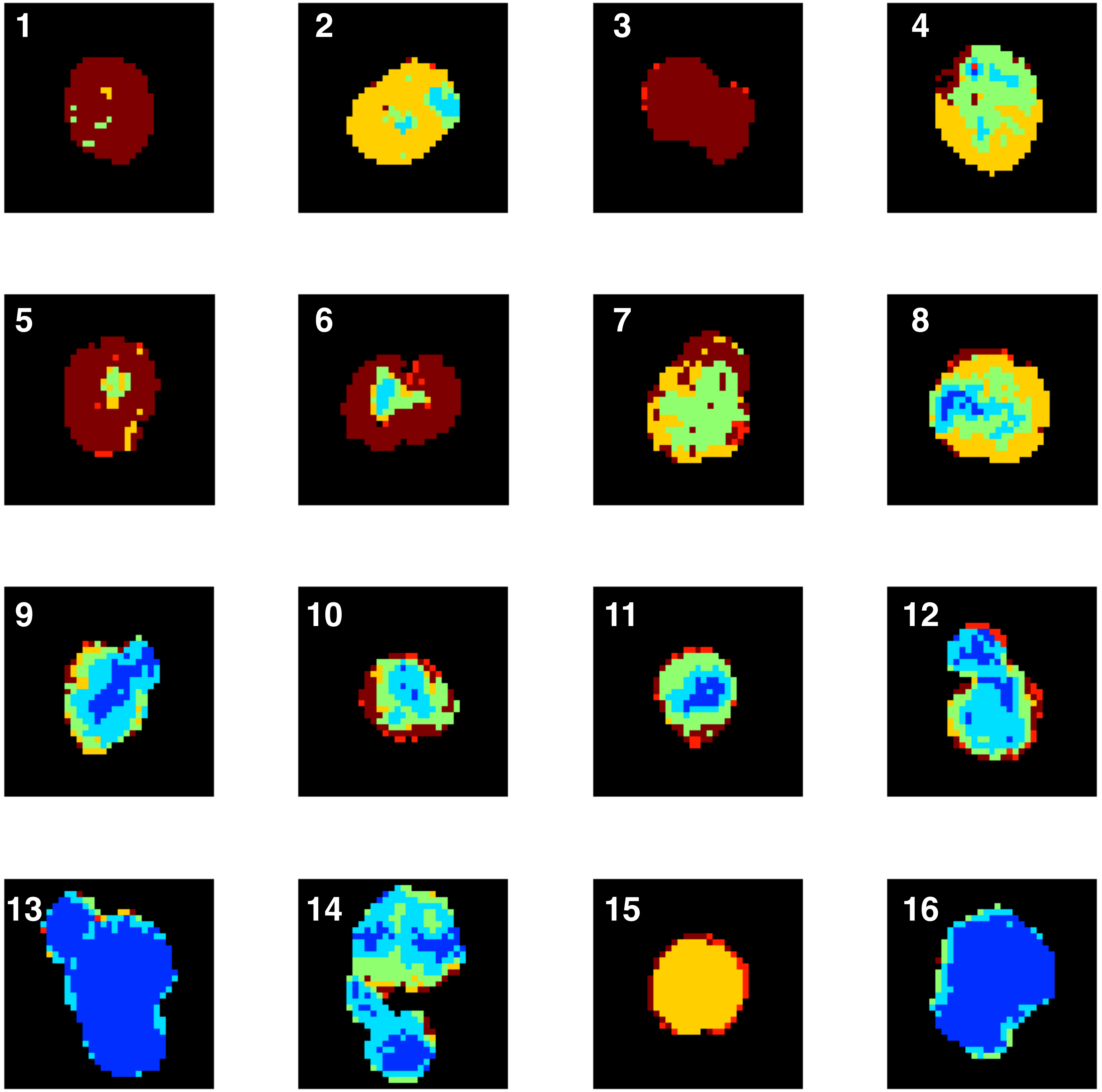

Fig. S9: Results from the optimized, data-driven (ODD) method: tumor region maps for central slices of all tumors, with colour coded cluster assignments to voxels. Rough rim-core structures are present in most tumors.

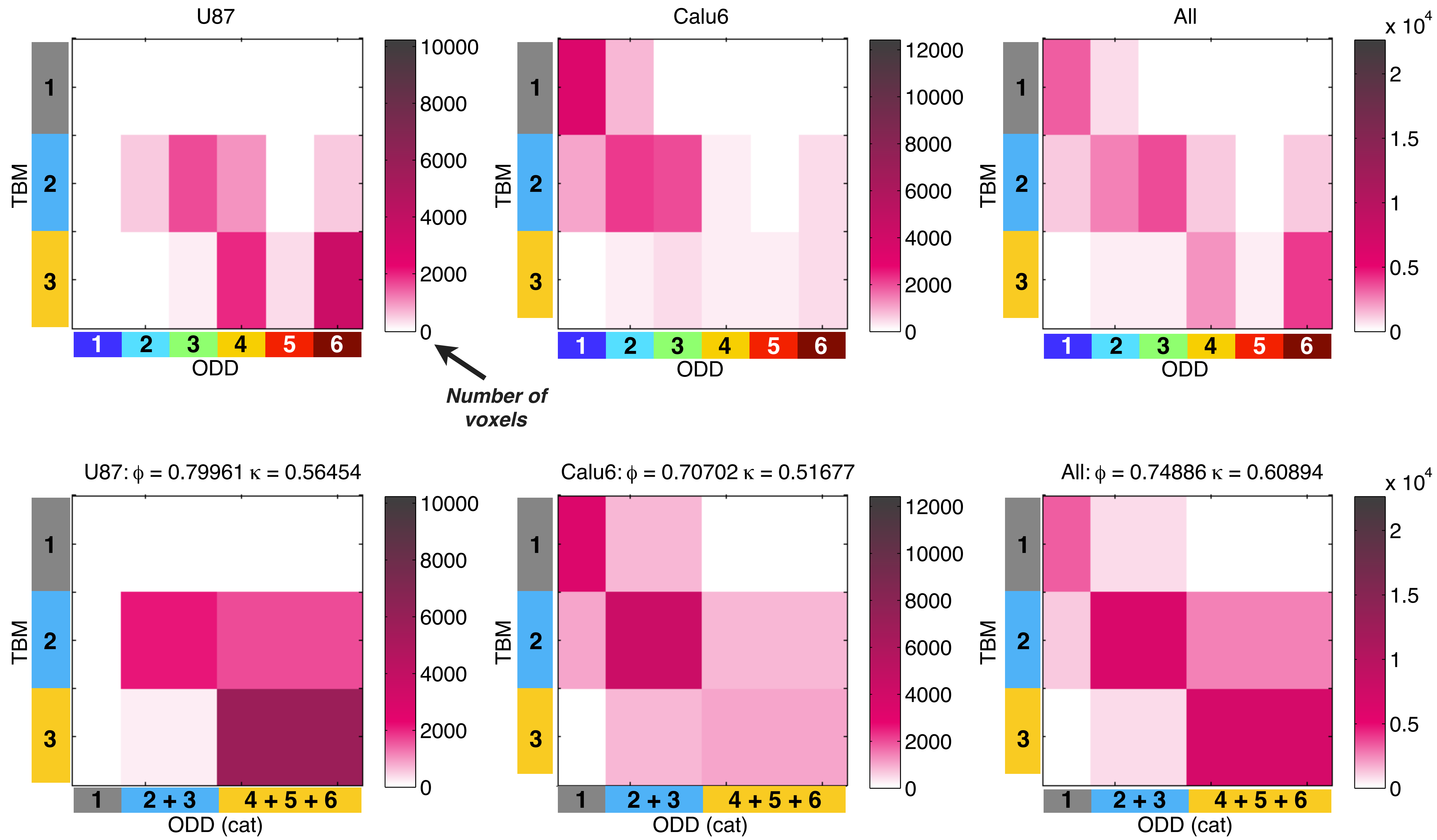

Fig. S10: Assignments grids showing how the previously published, threshold-based method (TBM) and our optimized, data-driven method (ODD) compare at assigning voxels to categories (top row). After concatentating the six categories from ODD into three classes, the bottom row shows assignment grids of TBM vs. ODD (cat), with proportional agreement (φ) and Cohen's kappa (κ) statistics calculated.

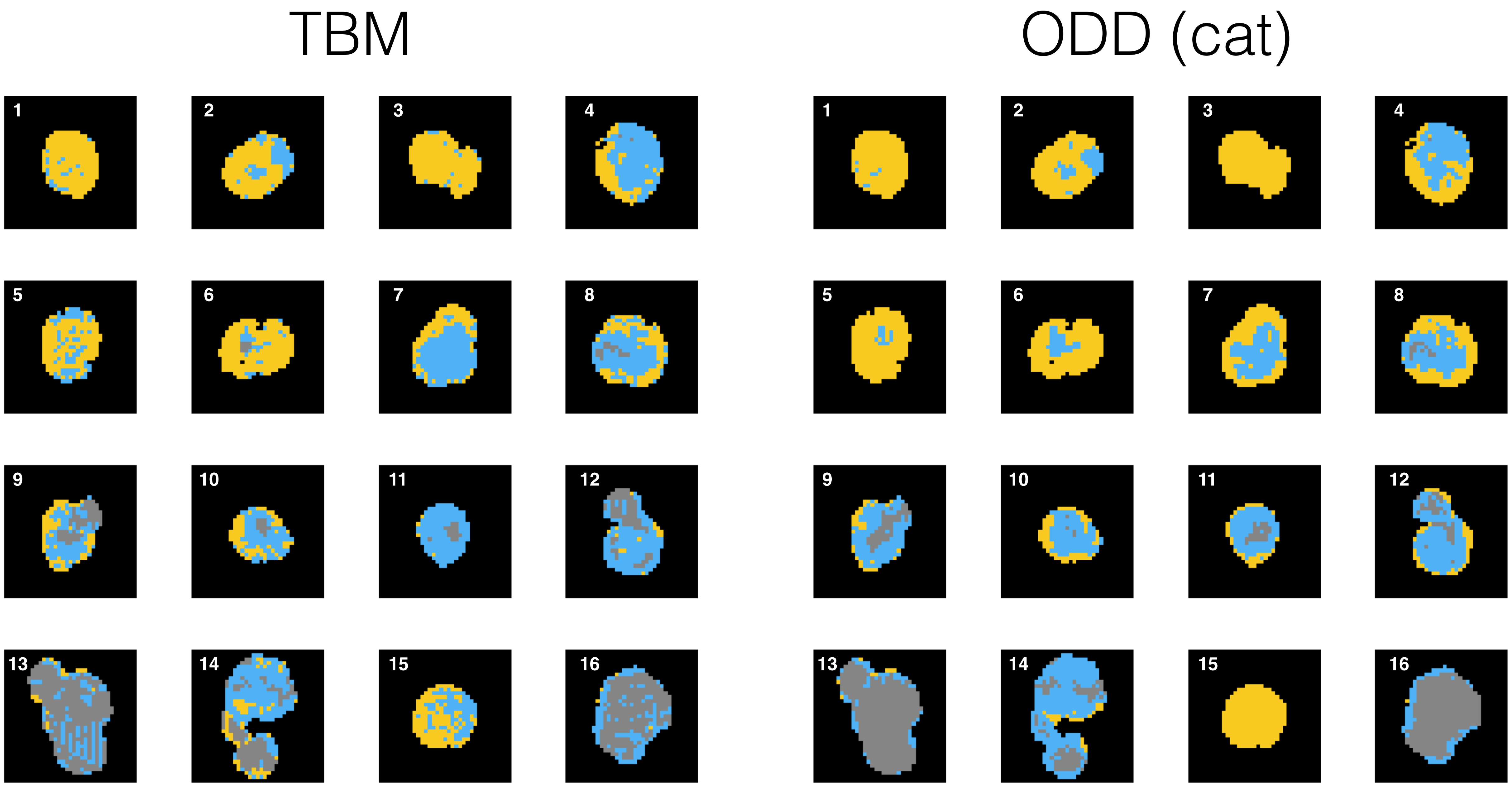

Fig. S11: Side by side comparison of region maps from the previously published, threshold-based method (TBM) and from the optimized, data-driven method concatenated into three classes (ODD (cat)).

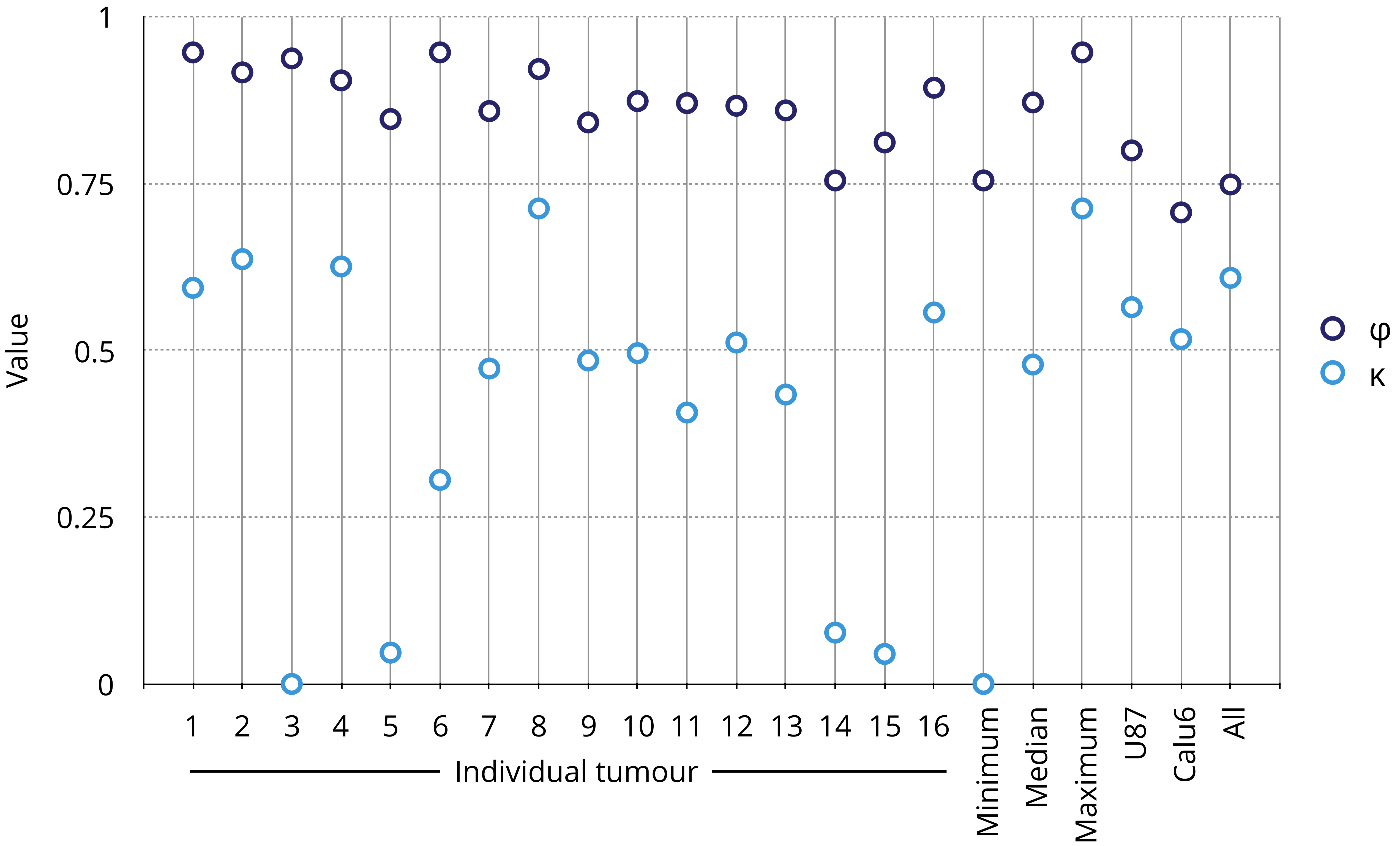

Fig. S12: Proportional agreement (φ) and Cohen's kappa (κ), calculated to rate agreement between the previously published, threshold-based method (TBM) and the optimized, data-driven (ODD) method. Values were calculated for individual tumors, the minimum, median, and max value highlighted, and group statistics were calculated for each tumor line and for the whole cohort.

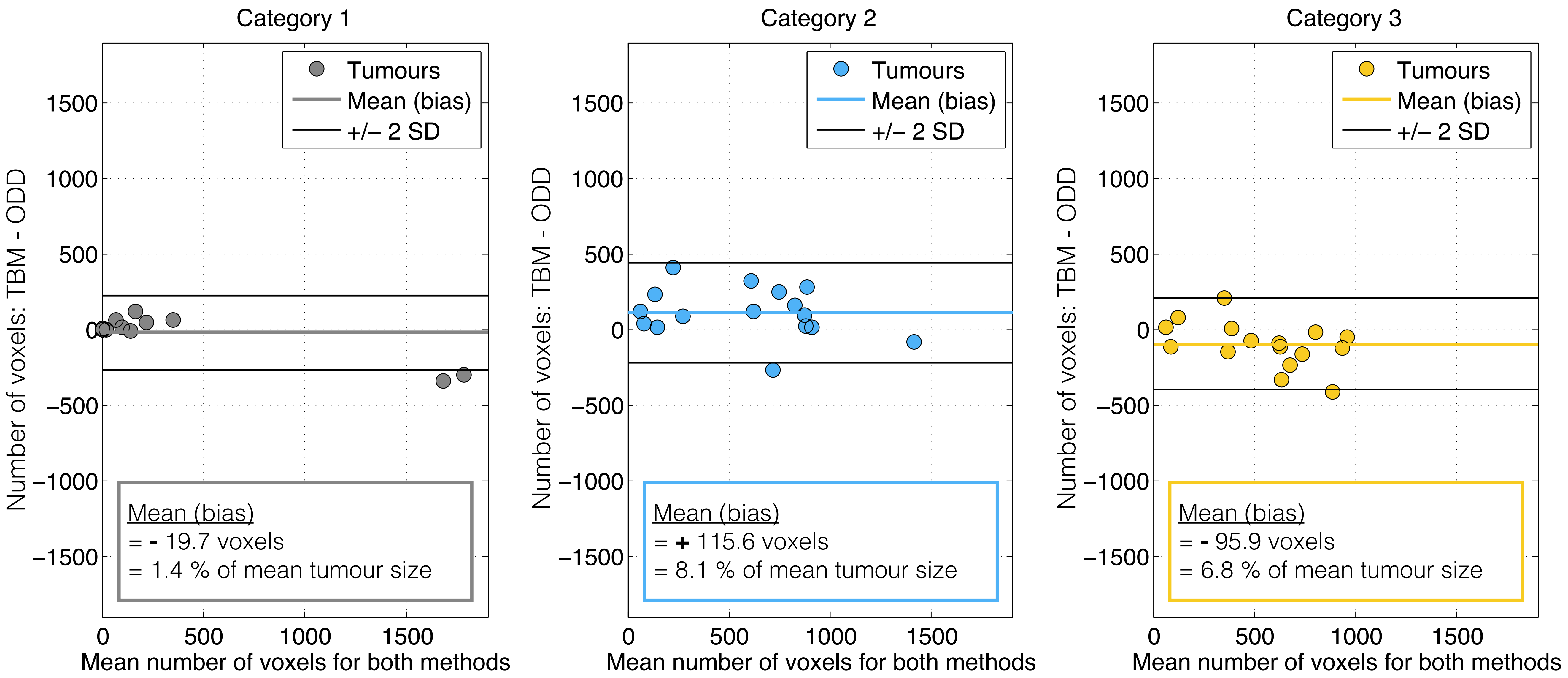

Fig. S13: Bland-Altman plots for the number of voxels in each of the three categories determined using the previously published, threshold-based method (TBM) and using the optimized, data-driven (ODD) method. For categories 1, 2, and 3, the bias in voxel categorisation (ODD - TBM) is -19.7, +115.6, and -95.9 voxels, corresponding to (-)1.4 %, 8.1 %, and (-)6.8 % of the mean tumor size (1419.2 voxels).
